# Effect of Mixture Variables on Durability for Alkali-Activated Slag Cementitious

**DOI:** 10.3390/ma11112252

**Published:** 2018-11-12

**Authors:** Chi-Che Hung, Yuan-Chieh Wu, Wei-Ting Lin, Jiang-Jhy Chang, Wei-Chung Yeih

**Affiliations:** 1 Institute of Nuclear Energy Research, Atomic Energy Council, Taoyuan 325, Taiwan; cchung@iner.gov.tw (C.-C.H.); ycwu@iner.gov.tw (Y.-C.W.); 2 Department of Civil Engineering, National ILan University, Yilan 260, Taiwan; 3 Department of Harbor and River Engineering, National Taiwan Ocean University, Keelung 202, Taiwan; jjc@mail.ntou.edu.tw (J.-J.C.); wcyeih@mail.ntou.edu.tw (W.-C.Y.)

**Keywords:** alkali-activated slag cementitious, absorption, resistivity, rapid chloride permeability test, carbonation rate

## Abstract

In this study, the influence of three mixture variables named Sand/Aggregate ratio, Liquid/Binder ratio, and Paste/Aggregate ratio on the cementitious properties were studied. The durability of cementitious including absorption, absorption rate, resistivity, rapid chloride permeability index, and carbonation rate were examined. Results showed that the alkali-activated slag cementitious has superior durability. The trends of influences on the composites properties for these three mixture variables are similar to those for the ordinary Portland cement concrete. It means that the experiences for making the ordinary Portland cement concrete should be able to be used for the alkali-activated slag cementitious. This paper also provides a lot of data for the alkali-activated slag cementitious for future development of the mix design.

## 1. Introduction

The environmental impact of the production of cement has prompted research into the development of alkali-activated cementitious using 100% replacement materials activated by alkali solutions. The higher emission of greenhouse gases into the atmosphere was mainly CO_2_ and it was globally estimated to be around 5%-to-8% of all such impact [1,2,3,4]. Cutting down on cement use would not only reduce CO_2_ emissions and slow down the greenhouse effect, but would also promote energy conservation. However, by-products used in cement-based composites must meet the requirements for strength and durability and its components should be obtained, produced, and used in an environmentally friendly manner [5]. The alkali-activated slag cementitious (AASC) attracted lots of attentions recently and was thought as the construction materials for this field [6] and it does not have much CO_2_ emission, it is then thought as a rising star in construction materials [7]. Besides, the alkali-activated cementitious has some properties and characteristic that is significantly better than ordinary Portland cementitious (OPC), such as lower heat of hydration and higher sulfate resistances [8]. Gluk-hovsky has been introduced AASC in 1958 [9] and it has the following superior properties: High compressive strength [10], good abrasion resistance, particularly when mixed with PTFE filler [11], excellent fire resistance than the OPC [12,13,14], good resistance to acid attack [15], solidification of radioactive waste [16], and so on.

While the development of AASC proceeded quickly in last decades, alkaline activators have a positive effect on the development of mechanical strengths of AASC [17,18]; however, the AASC mixture has some problems, which limited its application, such as rapid setting [19] and higher amount of autogeneous and drying shrinkage [20]. Shrinkage is well linked to the cracking tendency and consequently the durability related properties. According to previous studies, some alkaline activators were developed to improve the disadvantages. Malic acid or sodium chloride [21] and phosphoric acid [22] have been reported to have good retarding effects. The gypsum [23] and polypropylenglycol derivatives [24,25] can reduce the shrinkage. In addition, the AASC does not have a mix design procedure like the OPC. In References [26,27], the mix design methods named as the empirical mix design method, the experimental design method and particle packing theory are provided. The workability, strength development and mechanical properties for AASC have been reported in References [28,29] and it was completely developed. However, these data are not enough to build up a comprehensive understanding of the relationship between raw materials and durability of AASC. However, the literature on the application of the AASC durability is lacking a feasibility study on the various parameters of AASC mixtures. 

For AASC, usually three phases can be distinguished: The binder, aggregates, and the interfacial transition zone. The interfacial transition zone is the interface between the binders and aggregates and was the weakest phase in cement-based composites. It is sometimes recognized as a thin layer or the weaker binder phase and is thus controlled by the properties of binder. It was constituted by the cause of crack development and brought about material damage. However, the active mineral additives, such as AASC, are able to strengthen interfacial transition zone, change its morphology and reduce the size of microcracks occurring in this phase [30]. Improvement of the interfacial transition zone properties was positively influenced by AASC [31] due to the result of its homogenization and reducing the size of fracture occurring in this zone. The procedures also enhanced the lower permeability and higher durability. 

In this study, the influence of three parameters in mix design was examined with parameters as the Liquid/Binder ratio (referred to the water/cement ratio of the OPC), the Sand/Aggregate ratio, and Paste/Aggregate ratio for the evaluation of AASC properties. The fresh properties such as the workability, air content, and unit weight were tested; the mechanical properties such as the compressive strength, elastic modulus, and tensile splitting strength were conducted and evaluated. Through such a systematic study, the useful results can be provided for the future development of mix design of AASC.

## 2. Experimental

### 2.1. Materials

The ground granulated blast furnace slag with specific surface of 4000 cm^2^/g was used and its physical properties were listed in Table 1, which is produced by the CHC Resources Corporation (Kaohsiung, Taiwan). Crushed stones from Lanyang River (Yilan, Taiwan) were used as the coarse aggregate and the fineness modulus was 6.40. The river sands from Lanyang River in Taiwan were used as the fine aggregate and the fineness modulus was 2.59. The alkali activator was prepared by sodium hydroxide solution and sodium silicate (SiO_2_-37%, Na_2_O-17.7%, H_2_O-45.3%). To retard the fast setting behaviors of alkali-activated slag cementitious, the phosphoric acid was used as a setting retarder. The alkali activator was mixed to keep the following proportion: SiO_2_ = 100 g/L, Na_2_O = 100 g/L, and H_3_PO_4_ = 0.74 M.

All specimens were made into the cylindrical specimens (ϕ100 mm × 200 mm) and cured in the saturated lime-water at the temperature of 20 ± 2 °C until the test. Three specimens of each mixture were tested and the average was taken for each test. A standard deviation was controlled less than 10% for the tested results. Three parameters were chosen as Liquid/Binder ratio, Sand/Aggregate ratio and Paste/Aggregate ratio (where Liquid = alkali activator + phosphoric acid; Binder = slag; Aggregate = fine + coarse aggregate; and Paste = liquid + binder). Mix proportions of AASC are summarized in Table 2. It was fixed the Liquid/Binder ratio (L/B) and Paste/Aggregate ratio (P/A) then examined the effects of Sand/Aggregate ratio (S/A) as group I. For group II, the effects of Liquid/Binder ratio were mainly examined. For group III, the effects of Paste/Aggregate ratio were mainly examined. It was focused on the Sand/Aggregate ratio due to the weakest phase from fine aggregates. The mixtures were designed to confirm whether the Sand/Aggregate ratio was a key parameter for AASC composites.

### 2.2. Experiments Conducted

**Figure 1:**
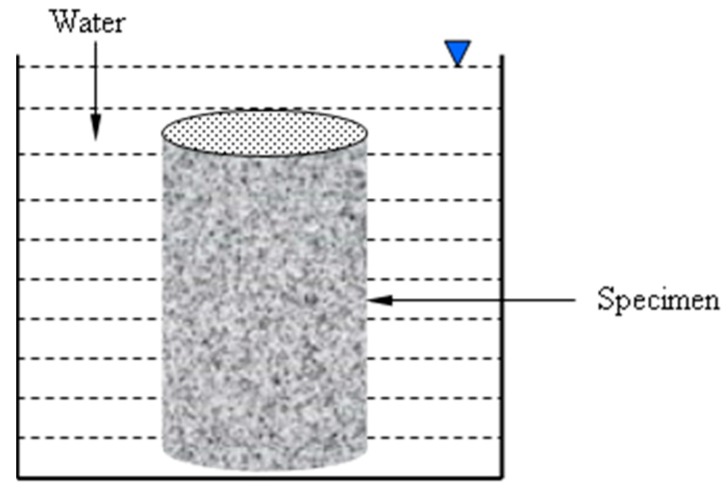
Schematic diagram of absorption test.

The influence of three parameters on the durability was examined. The water absorption was calculated according to the standard procedure American Society for Testing and Materials (ASTM) C642. In this test, the water absorbed by an oven-dried specimen is measured after 24 h immersion. The set-up of absorption test is illustrated in Figure 1 and the absorption was calculated as follows:

(1) $$\mathrm{Absorption}\left(\%\right)=\frac{W_{i}-W_{d}}{W_{d}}\times 100\%$$

where *W_i_* is the weight of specimen after 24 h immersion and *W_d_* is the weight of specimen on oven-dried condition.

The resistivity was measured by four-electrode resistivity instrument following the specification of ASTM WK37880 [32]. The apparent resistivity ρ is given by the following expression:

(2) $$\rho =\frac{2\pi aV}{I}$$

where *a* is probe spacing, *V* is the voltage between the inner electrodes and *I* is the current between the outer electrodes. In this study, the probe spacing used was 5 cm.

The carbonation rate was measured by a high-pressure carbonation instrument. The conditions of this test were: CO_2_ concentration = 100%, pressure = 15 atm, temperature = 25 ± 2 °C, and relative humidity = 70 ± 5%. The carbonation rate was measured in agreement with phenoftalein method on the split surface of the specimens.

**Figure 2:**
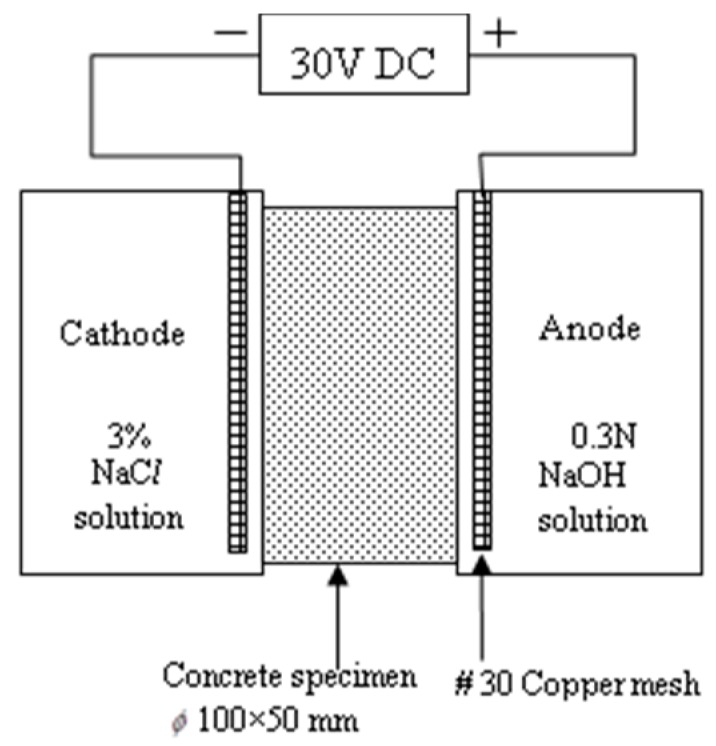
Schematic illustration of rapid chloride penetration test.

The dimensions of the resistivity, absorption, and carbonation test were ϕ100 mm × 200 mm. The resistance to chloride ion penetration was determined in accordance with the standard procedure ASTM C1202, and the specimen diameter (D) and height (H) of the specimens were 100 and 50 mm, respectively. The lateral surface of the specimen was coated with epoxy, and the specimen was placed in a vacuum desiccator with air pressure less than 1 mm-Hg (133 MPa) for 3 h. The specimen was immersed in deaerated water and the vacuum level was maintained for 1 h. The specimen was then removed and immersed in water for 18 h. A schematic setup of the rapid chloride penetration test is illustrated in Figure 2.

Scanning electron microscopy (SEM, HITACHI Ltd., Tokyo, Japan) analysis was conducted on representative samples of 1 mm × 1 mm × 1 mm, in accordance with ASTM C1723. The samples with saw marks are removed using 600 grit followed by 1000 grit sandpaper. Dry the samples in a forced draft oven at a temperature of 100 ± 5 °C. Non-conductive samples can be coated with a thin layer of gold or carbon and thus a relatively high resolution can be obtained from secondary electrons.

## 3. Results and Discussions

### 3.1. Water Absorption

**Figure 3:**
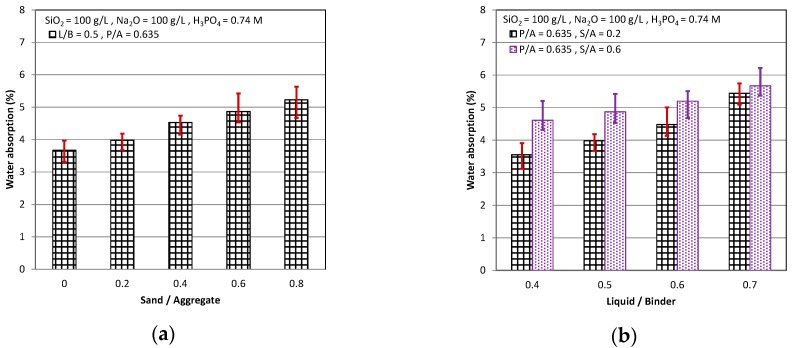

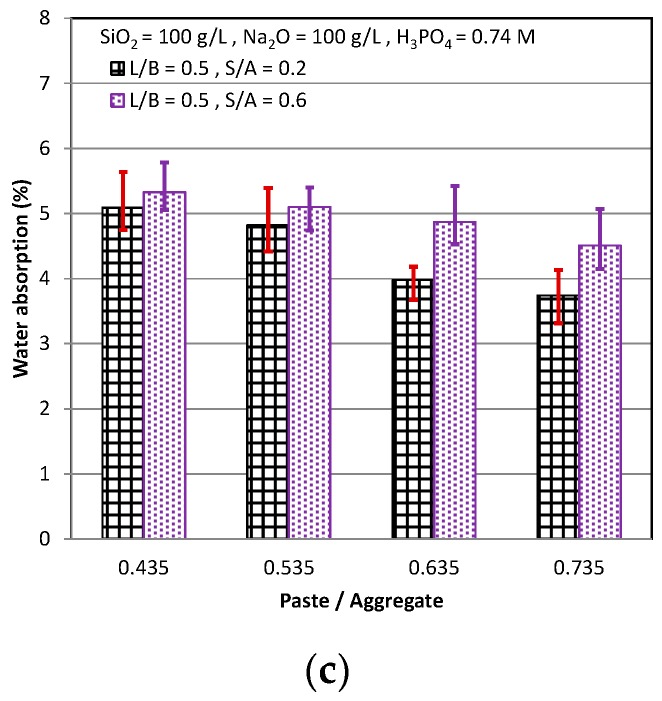
Water absorption: (**a**) P/A = 0.635, L/B = 0.5; (**b**) P/A = 0.635, S/A = 0.2 & 0.6; and (**c**) L/B = 0.5, S/A = 0.2 & 0.6.

In Figure 3, it can be seen that as the curing age increased, the water absorption decreased. The cementitious became more compact with the curing age because of the hydration reaction of the slag and the alkali activator, and it had a worse ability to absorb the water into the smaller pores. The water absorption increased as the S/A increased, as shown in Figure 3a. It is indicated that as the S/A increased because of that the sand had a higher specific surface area than the coarse aggregate, which resulted in the much weaker interface between the aggregate and the paste. Consequently, the large amount of water was absorbed into the weak interface as the S/A increased. In Figure 3b, it can be observed that increasing L/B increased the water absorption. We all know that as the water/cement ratio was higher, there was large water in the fresh cementitious. After the hydration between the slag and the alkali activator, the un-hydrated water will be absorbed. Therefore, a considerable amount of the pores were formed from the evaporated water. The L/B in the AASC was similar to the water/cement ratio in the OPC, so that as the L/B was higher, a great quantity of the pores was generated which increased water absorption. The influence of P/A on the water absorption is shown in Figure 3c. The higher water absorption in the OPC resulted largely from great amounts of gel pores that were formed from the un-hydrated water in the paste. As the P/A increased, the water absorption increased in the OPC. However, the gel pores in AASC were more compact than that in the OPC, and had a worse ability on the water absorption than that in the interface between the pastes and the aggregates, which is consistence with the previous result, that was weaker, between the aggregates and pastes [9,33]. Consequently, as the Paste/Aggregate ratio increased in the AASC, the water absorption decreased.

### 3.2. Absorption Rate

**Figure 4:**
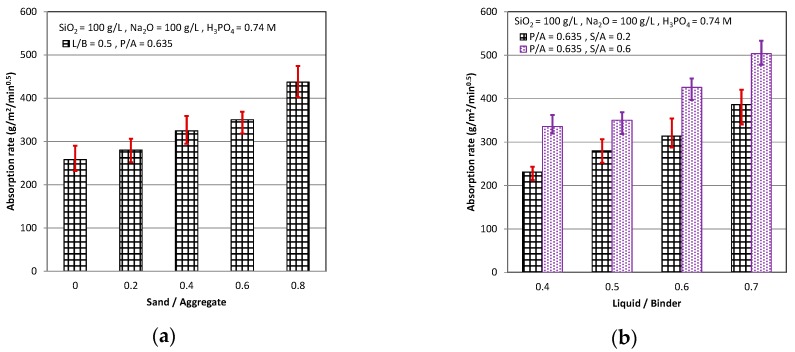

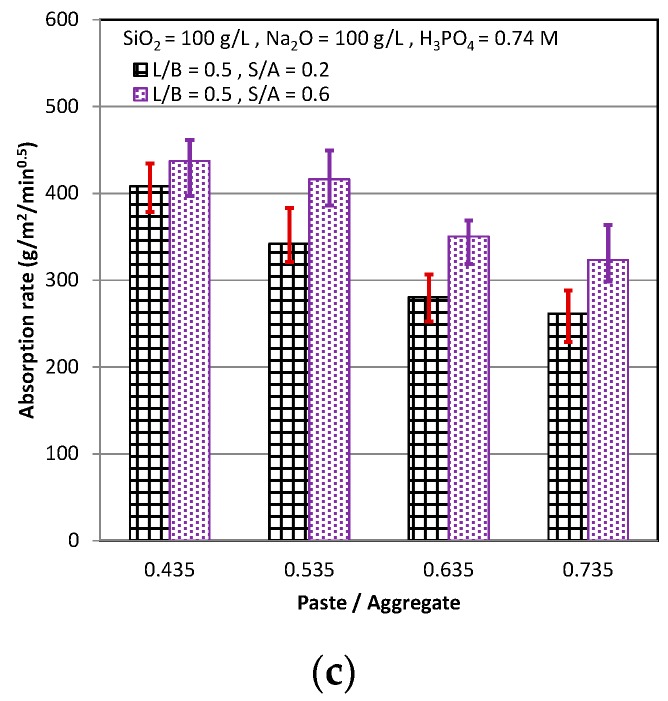
Absorption rate: (**a**) P/A = 0.635, L/B = 0.5; (**b**) P/A = 0.635, S/A = 0.2 & 0.6; and (**c**) L/B = 0.5, S/A = 0.2 & 0.6.

The result of absorption rate test, which measured the weight of water on the capillary suction and then calculated the rate of the water absorption, is shown in Figure 4. The absorption rate increased when the S/A and L/B increased, as shown in Figure 4a,b, and decreased when the P/A increased, as shown in Figure 4c. The trends of the absorption rate were similar to that of the water absorption, although the measure methods and meaning of the absorption rate were different from that of the water absorption. Roughly speaking, the durability, which can prevent the steel from corroding by the O_2_ and H_2_O in the air, depended on the degree of the compactness of the cementitious. As the water absorption and absorption rate decreased, the amounts of the holes and the weak interface in the cementitious were less, and the cementitious had better durability. For this reason, as the S/A and L/B decrease and the P/A increased, it could let the AASC have the better durability.

### 3.3. Resistivity

**Figure 5:**
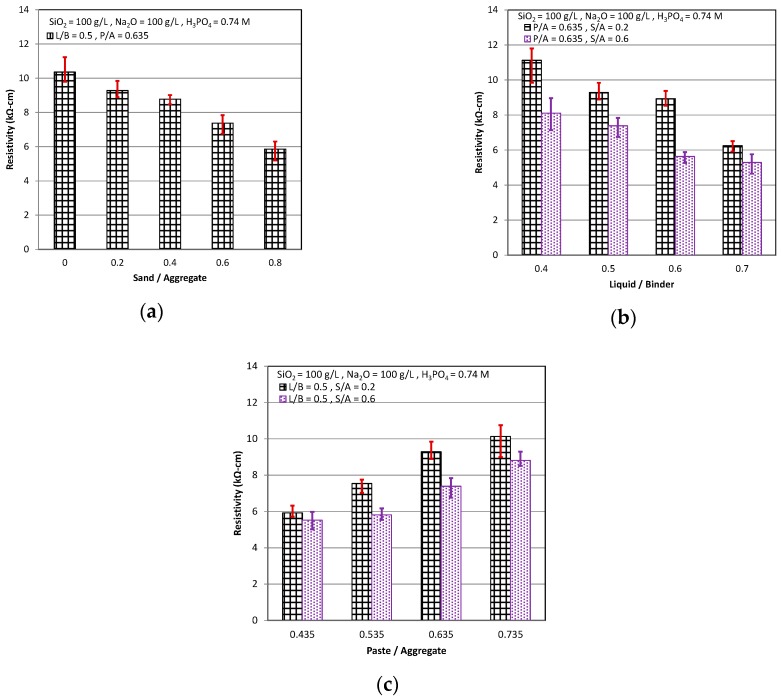
Resistivity: (**a**) P/A = 0.635, L/B = 0.5; (**b**) P/A = 0.635, S/A = 0.2 & 0.6; and (**c**) L/B = 0.5, S/A = 0.2 & 0.6.

The test result of the resistivity is shown in Figure 5. It can be seen that the resistivity became higher with the curing age. The resistivity must be measured by electric conduction that resulted from the movement of the ions in the electrolytic liquid. The electrolytic liquid was present in the pores of the cementitious or in the transition zone of the weak interface between the paste and the aggregate. Thus, it means that there existed the fewer pores in the cementitious as the resistivity became higher. Just like this, as the S/A and L/B decreased and the P/A increased, the pores in the cementitious or the transition zone of the weak interface were fewer, which caused the pore liquid to have a lower electric conduction by the fewer ions in the electrolytic liquid of the cementitious and consequently the cementitious had the higher resistivity (see Figure 5). 

### 3.4. Rapid Chloride Permeability Test

**Figure 6:**
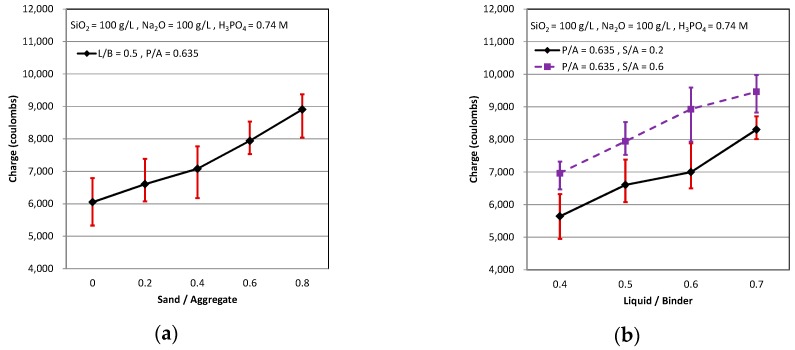

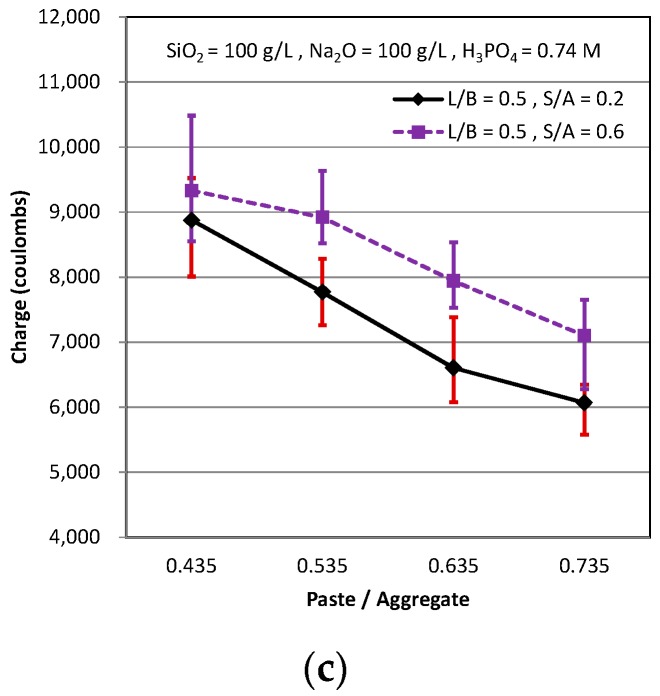
RCPT: (**a**) P/A = 0.635, L/B = 0.5; (**b**) P/A = 0.635, S/A = 0.2 & 0.6; and (**c**) L/B = 0.5, S/A = 0.2 & 0.6.

The alkaline environment provided by the cementitious would produce the passive protective membrane that prevents the reinforcing bar from corrosion, but the existence of the chlorine ion would destroy the passive protective membrane and let the reinforcing bar to corrode easily. Rapid chloride permeability test (RCPT) is one kind of method that can measure the permeability of the chloride ion in the cementitious and estimate the resistance from the chloride ion through the cementitious in the short-term. The influence of three mix design parameters on the RCPT is shown in Figure 6. As curing time was longer, the through electric charge was less, and it represented that the cementitious owned the better resistance from the chloride ion through the cementitious. 

As shown in Figure 6a, when the S/A was higher, the through electric charge was higher. This means that the proportions of the sand were more, and it is easy to be permeated by the chloride ion in the cementitious. It is because that the content of the sand was more, there was the more interface between the paste and the aggregate too. Accordingly, the chloride ion permeates into the interface between the paste and the aggregate and passes the cementitious easily. It can be seen that the through electric charge was high as L/B were high, as shown in Figure 6b. It known that the Liquid/Binder ratio of AASC was similar to the Water/Cement ratio of OPC. As Water/Cement ratio was higher, the cementitious was made from the inferior paste, and so the cementitious had a worse resistance by the chloride ion. The through electric charge of RCPT decreased as the P/A increased (see Figure 6c). The trend of AASC is dissimilar to that of OPC. As the cement paste of the OPC was more, the cementitious was less compact, and it owned the worse ability to resist the chlorine ion passed through the cementitious. It may be due to its less porous micro-structures, which is consistence with the previous study [34]. However, the cementitious had a better ability to resist the chloride ion as the alkali-activated slag paste of AASC increased. It represented that the chlorine ion permeated and passed through the OPC more easily than through the AASC. In addition, the alkali-activated slag paste was more compact than the interface between the paste and aggregate. Therefore, as the paste increased and the aggregate decreased, the resistance of the chlorine ion in the AASC was better. 

### 3.5. Carbonation Rate

**Figure 7:**
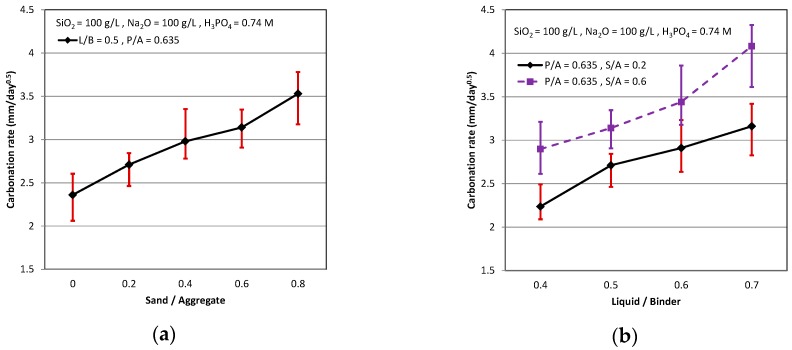

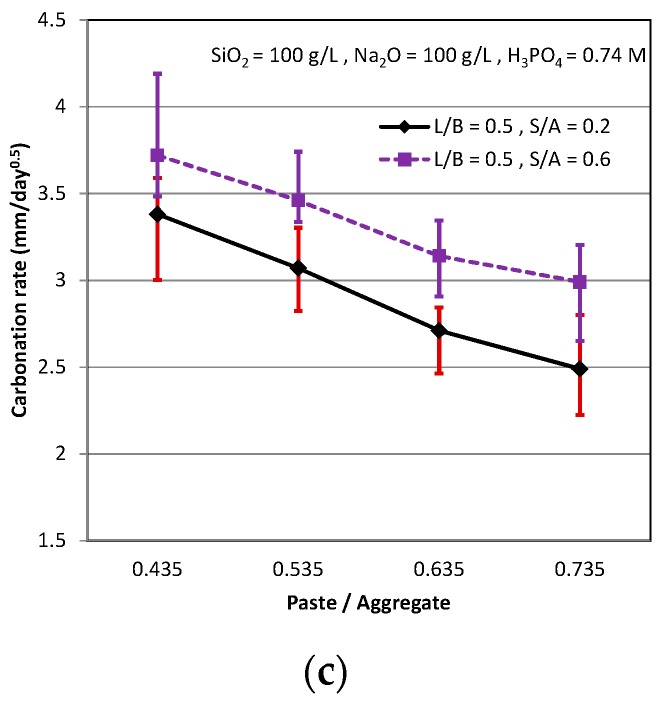
Carbonation rate (pH indicator): (**a**) P/A = 0.635, L/B = 0.5; (**b**) P/A = 0.635, S/A = 0.2 & 0.6; and (**c**) L/B = 0.5, S/A = 0.2 & 0.6.

The existence of passive protective membrane on the reinforcing bar in the alkaline environment like the cementitious can be found, but the disappearance of passive protective membrane on the reinforcing bar in the un-alkaline environment, like the cementitious carbonated. Thus, the reinforcing bar corroded easily in the disappearance of passive protect membrane on it as the cementitious suffered from CO_2_ and became neutral from alkaline. The carbonation rate, measured by the pH indicator and phenolphthalein indicator, is shown in Figure 7. It can be seen that the carbonation rate by the pH indicator was higher than by the phenolphthalein indicator. The phenolphthalein indicator only can determine that the pH is higher than 9.2 or not, but the pH indicator can accurately determine that the pH is higher than 11 or not from the carbonated cementitious. Therefore, the carbonation rate by the pH indicator was more accurate and higher than by the phenolphthalein indicator. 

The influence of three mix design parameters on the carbonation rate by the pH indicator is illustrated in Figure 7. The carbonation rate increased as the S/A and L/B increased, as shown in Figure 7a,b. The trends of carbonation rate in the AASC were similar to the trends in the OPC, because, as the sand increased or the liquid increased, it caused both the cementitious incompact. The carbonation rate decreased as the P/A increased, as shown in Figure 7c. We know that as the amount of the cement increased, the pH value was 11 stably in the OPC, and it was difficult to be carbonated. The alkali-activated slag paste in the AASC, which was similar to the cement paste in the OPC, can not only let the cementitious be carbonated with difficulty, but also contributes more durability than the paste of OPC.

### 3.6. SEM Observations

**Figure 8:**
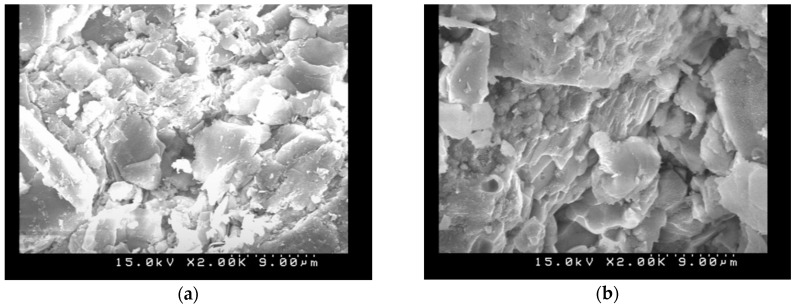

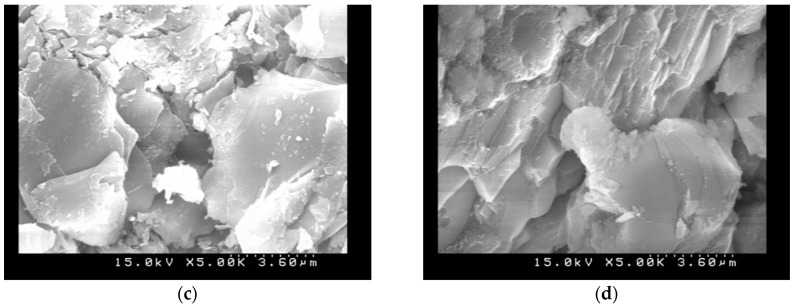
SEM images (S/A = 0.2, L/B = 0.5, P/A = 0.435): (**a**) without carbonation (2000×); (**b**) with carbonation (2000×); (**c**) without carbonation (5000×); and (**d**) with carbonation (5000×).

**Figure 9:**
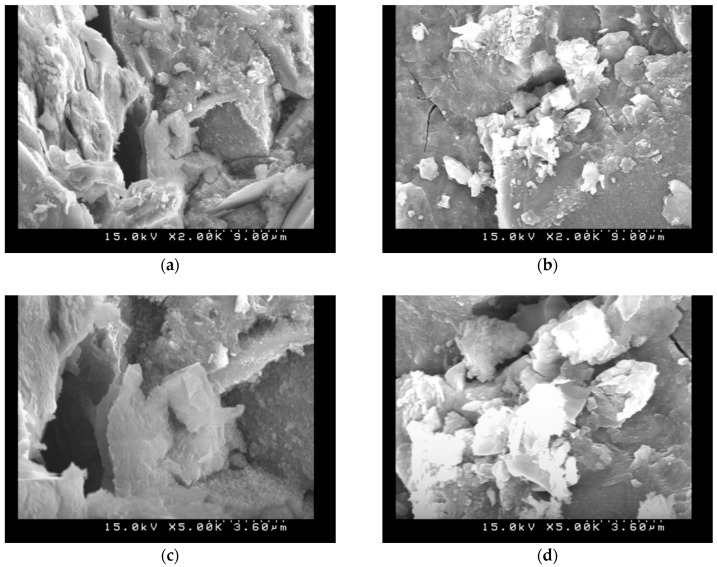
SEM images (S/A = 0.2, L/B = 0.5, P/A = 0.535): (**a**) without carbonation (2000×); (**b**) with carbonation (2000×); (**c**) without carbonation (5000×); and (**d**) with carbonation (5000×).

**Figure 10:**
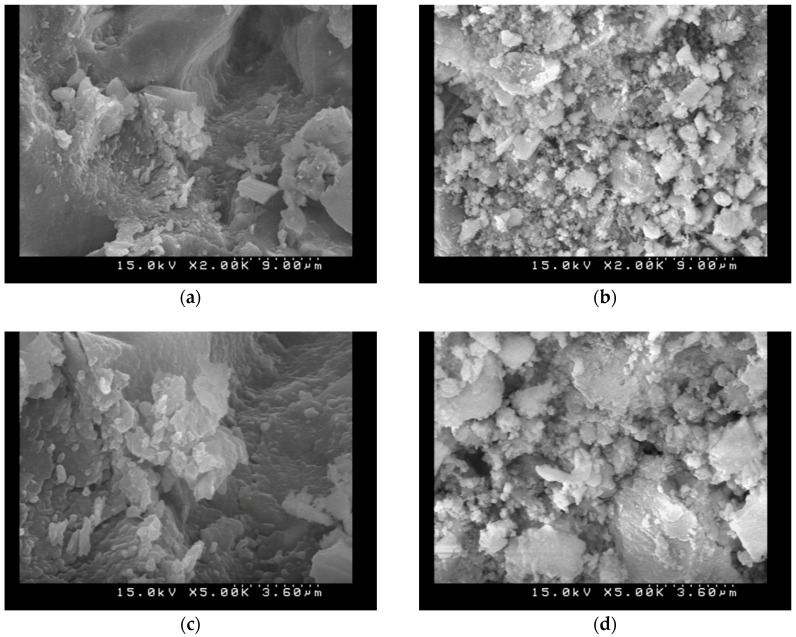
SEM images (S/A = 0.2, L/B = 0.5, P/A = 0.735): (**a**) without carbonation (2000×); (**b**) with carbonation (2000×); (**c**) without carbonation (5000×); and (**d**) with carbonation (5000×).

To evaluate the effects of the carbonation on the microstructure of the AASC specimens, SEM observations before and after carbonation tests for different P/A were conducted and the specimens had finer pore structures, as illustrated in Figure 8, Figure 9 and Figure 10. It indicated that the granules of the source material constitute a stratified structure and are bound with each other in amalgamation, leaving much ruggedness and porosity. As carbonation progressed, however, many acicular materials and hydrations were created in the porosity. These acicular structures entangle with each other, and by filling up the empty spaces. Carbonated AASC specimens with lower P/A ratio were shown to have significant disorganization and disintegration of its microstructure. It is also observed that the carbonated AAS with higher P/A ratio with smooth and compact surfaces are disintegrated and separated or that some adhered to the surface, increasing the amount of porosity. The reduced concentration of free alkalis in the pore solution in AASC specimens makes these pastes more vulnerable to carbonation, as the dissolved CO_2_ in pore solution will react with the hydrated binding phases, inducing a notable increase in the microporosity [35,36]. The hardening paste by reaction with the calcium released by the slag and this early-age formation of alkali carbonates is accompanied by early formation of calcium silicate hydrate or calcium alumina silicate hydrate as strength-giving products. The calcium carbonate phases formed at an early age remain stable in the reaction product assemblage. Besides, the water-soluble carbon dioxides formed carbonate ions and then the calcium carbonates reacted from carbonate ions with alkali solutions [19,37]. Carbonization may still be caused by the alkalinity in the pore water and this alkali may be provided by the unreacted alkali solutions, not provided by the calcium hydroxides, which is consistent with previous studies [19,37,38].

### 3.7. Performance Indicator

The comparison of fresh properties and durability for the three mix design parameter of AASC composites is summarized in Table 3. It indicated that the trends of fresh concrete properties in AASC were similar to OPC; however, some trends of durability were different from OPC. The performance indicators in OPC might not be suitable as the indicators in AASC. With the increase of S/A as well as with the decrease of L/B or P/A, the slump significantly decreased for each mixture of OPC and AASC composites. For the durability, the OPC and AASC composites had better durability with the decrease of S/A or L/B. Conversely, the OPC composites with the increase of P/A had lower durability, but this trend reverses for AASC. AASC was attributed to the strength development resulted in denser pastes, which is superior to the OPC pastes and the interfacial transition zone is the interface between the binders and aggregates. The microstructure properties and durability in OPC composites are strongly dependent on the improvement of the interfacial transition zone properties, resulting in the poor durability as the increase of P/A. Significant improvement in microstructure properties of the AASC composites has better performance than the improvement in interfacial transition zone. With the increase of P/A in AASC composites, the decrease as the amount of the interface between the binders and aggregates resulting in denser micro-structures. However, the expresses of OPC is crosscurrent.

## 4. Conclusions

In this paper, the influence of three parameters were investigated, S/A, L/B, and P/A on the durability of AASC. It can be concluded that the L/B no longer plays as the major factor affecting the durability. The durability of AASC are strongly dependent on the activator included type and concentration. Instead, the S/A and P/A are more significant. For OPC, increasing P/A reduces the durability. However, the trends in AASC composites reversed because the pastes now were no longer the weakest phase in the interfacial transition zone between the binders and aggregates. The pastes of AASC had better strength and denser micro-structures than that of OPC. However, it has a weaker interface between the aggregate and the paste. The results indicated that the sand (fine aggregates) had a higher specific surface area than the coarse aggregate. Higher surface area provides the weaker interface between the aggregate and the paste. In addition, carbonated AASC specimens with lower P/A ratio were showed significant disorganization and disintegration of its microstructure.

## Figures and Tables

**Table 1:** The chemical and physical properties of slag.

Item	Composition	Value
The main chemical composition of slag (By weight percentage)	SiO_2_ (%)	33.87
	Al_2_O_3_ (%)	14.42
	Fe_2_O_3_ (%)	0.69
	CaO (%)	39.54
	MgO (%)	5.35
	SO_3_ (%)	2.47
	Basicity coefficient Kb = (CaO + MgO)/(SiO_2_ + Al_2_O_3_)	0.93
Physical properties	Specific weight	2.90
	Ignition loss (%)	0.28
	Fineness (m^2^/kg)	383

**Table 2:** Mixture design.

Group	S/A	L/B	P/A	Air Content (%)	Liquid (kg/m^3^)	Binder (kg/m^3^)	Fine Aggregate (kg/m^3^)	Coarse Aggregate (kg/m^3^)	Slump (mm)
**I**	**0**	**0.5**	**0.635**	1.8	285	570	0	1346	220
	**0.2**	**0.5**	**0.635**	2.0	284	568	268	1073	190
	**0.4**	**0.5**	**0.635**	2.3	283	566	534	802	160
	**0.6**	**0.5**	**0.635**	2.4	282	564	799	533	130
	**0.8**	**0.5**	**0.635**	2.6	281	562	1062	265	110
**II**	**0.2**	**0.4**	**0.635**	2.5	249	622	274	1098	70
	**0.2**	**0.5**	**0.635**	2.0	284	568	268	1073	190
	**0.2**	**0.6**	**0.635**	1.5	314	523	263	1054	230
	**0.2**	**0.7**	**0.635**	1.2	339	484	259	1036	260
	**0.6**	**0.4**	**0.635**	2.8	247	619	818	546	50
	**0.6**	**0.5**	**0.635**	2.4	282	564	799	533	130
	**0.6**	**0.6**	**0.635**	1.9	311	519	784	523	170
	**0.6**	**0.7**	**0.635**	1.5	336	481	772	515	230
**III**	**0.2**	**0.5**	**0.435**	2.1	230	459	317	1267	40
	**0.2**	**0.5**	**0.535**	2.1	259	518	290	1162	80
	**0.2**	**0.5**	**0.635**	2.0	284	568	268	1073	190
	**0.2**	**0.5**	**0.735**	1.8	306	612	250	998	220
	**0.6**	**0.5**	**0.435**	2.7	227	455	941	627	20
	**0.6**	**0.5**	**0.535**	2.6	257	514	864	576	50
	**0.6**	**0.5**	**0.635**	2.4	282	564	799	533	130
	**0.6**	**0.5**	**0.735**	2.3	303	607	743	495	150

**Table 3:** Comparison of fresh properties and durability for three mix design parameters.

Parameters	Fresh Properties		Durability				
	Air Content	Slump	Absorption	Absorption Rate	Resistivity	Rapid Chloride Permeability Index	Carbonation Rate
**Increase S/A**	+	−	+	+	−	+	+
**Increase L/B**	−	+	+	+	+	+	+
**Increase P/A**	−	+	−	−	+	−	−

Note: +: increment −: reduction.
